# Excess Body Mass Index Loss at 3 Months: A Predictive Factor of Long-Term Result after Sleeve Gastrectomy

**DOI:** 10.1155/2017/2107157

**Published:** 2017-01-29

**Authors:** Guillaume Philouze, Eglantine Voitellier, Laurence Lacaze, Emmanuel Huet, Antoine Gancel, Gaëtan Prévost, Michael Bubenheim, Michel Scotté

**Affiliations:** ^1^Department of Digestive Surgery, Rouen University Hospital, 1 rue de Germont, 76031 Rouen, France; ^2^Department of Endocrinology, Bois-Guillaume Hospital, 76230 Bois-Guillaume, France; ^3^Department of Biostatistics, Rouen University Hospital, 1 rue de Germont, 76031 Rouen, France

## Abstract

*Introduction.* Laparoscopic Sleeve Gastrectomy (SG) is considered as successful if the percentage of Excess Body Mass Index Loss (% EBMIL) remains constant over 50% with long-term follow-up. The aim of this study was to evaluate whether early % EBMIL was predictive of success after SG.* Methods.* This retrospective study included patients who had SG with two years of follow-up. Patients had follow-up appointments at 3 (M3), 6, 12, and 24 months (M24). Data as weight and Body Mass Index (BMI) were collected systematically. We estimated the % EBMIL necessary to establish a correlation between M3 and M24 compared to % EBMIL speeds and calculated a limit value of % EBMIL predictive of success.* Results.* Data at operative time, M3, and M24 were available for 128 patients. Pearson test showed a correlation between % EBMIL at M3 and that at M24 (*r* = 0.74; *p* < 0.0001). % EBMIL speed between surgery and M3 (*p* = 0.0011) was significant but not between M3 and M24. A linear regression analysis proved that % EBMIL over 20.1% at M3 (*p* < 0.0001) predicted a final % EBMIL over 50%.* Conclusions.* % EBMIL at M3 after SG is correlated with % EBMIL in the long term. % EBMIL speed was significant in the first 3 months. % EBMIL over 20.1% at M3 leads to the success of SG.

## 1. Introduction

Laparoscopic Sleeve Gastrectomy (SG), initially considered as the first step of bariatric surgery for super-obese patients [1, 2], is now considered as a single procedure due to its effectiveness on the resolution of comorbidities and weight loss [3–5]. This attitude has been confirmed by three international consensus summits [6–8].

SG is a restrictive bariatric surgery in which two-thirds of the stomach is resected. It has been proven to be as efficient on weight loss as Laparoscopic Adjustable Gastric Band (LAGB) or RYBP. According to the International Federation for Obesity and Metabolic Disorders (IFSO), the procedure is considered successful if the percentage of Excess Body Mass Index Loss (% EBMIL) remains constant over 50% with long-term follow-up. In most studies, % EBMIL following SG ranges between 45 and 65% [5, 9, 10].

The main predictive operative factors of success that have been studied are the calibre of the bougie, the distance of the resection to the pylorus and the volume, and the estimation of the gastric reservoir volume after surgery [11–13]. Clinical characteristics such as initial weight and BMI, age, comorbidities, preoperative weight loss, and personality disorders have also been described as predictors of final results [14].

It is now well established that % EBMIL is major during the first 3 months after surgery and slowly rises during the following months until 2 years [15].

The objective of this present study was to determine whether the % EBMIL at 3 months was predictive of the long-term result, 2 years after SG.

## 2. Materials and Methods

### 2.1. Patient Selection

This retrospective observational study was performed in the Department of Digestive Surgery of a tertiary care hospital. This study included all consecutive patients who had SG procedure, with a complete follow-up of more than 2 years.

### 2.2. Inclusion Criteria

All patients included fulfilled the criteria of the January 2009 French National Health Authority (Haute Autorité de Santé) for bariatric surgery. SG was indicated in patients between 18 and 60 years with a BMI higher than 40 kg/m^2^ or between 35 and 40 kg/m^2^ with severe comorbidity. We selected all patients who achieved 2 years of follow-up.

### 2.3. Exclusion Criteria

Patients younger than 18 or older than 60 years or patients who did not complete the preoperative workup and follow-up until 2 years were excluded. Patients with psychiatric disorder, alcohol, or drug dependence were also considered as not suitable for SG.

### 2.4. Operative Technique

All procedures were carried out by three surgeons with considerable experience in SG since 2005. The dissection of the gastric greater curve started at approximately 6 cm from the pylorus, proceeding upward until the angle of His using either radiofrequency or tissue sealing device. The SG was created using a linear stapler applied alongside a 36 Fr bougie strictly positioned against the lesser curve. Then the resected stomach was extracted. A methylene blue test was systematically performed to check for leaks before eventual reinforcement using staples or ligatures.

### 2.5. Postoperative Follow-Up

The following preoperative data were collected: date of birth, age, sex, weight, height, and major comorbidities (hypertension, sleep apnea, type 2 diabetes mellitus (T2DM), dyslipidemia, and other obesity-related diseases). Thus, BMI and ideal weight according to Lorentz formula were calculated for all patients (Table 1).

After SG, patients had regular follow-up at 3, 6, 12, and 24 months (M3, M6, M12, and M24). At each appointment, weight and comorbidities were noted, and we calculated the BMI and the percentage of excess weight loss (% EWL) and total weight loss (% TWL) using the ideal weight for all patients as a standard. After the M24 appointment in our Department of Digestive Surgery, follow-up was continued by the patient's General Practitioner.

### 2.6. Definition of Comorbidities

According to the HAS, hypertension was defined as systolic blood pressure ≥ 140 mmHg and/or diastolic blood pressure ≥ 90 mmHg, confirmed by two measurements after 3 successive consultations over a period of 3 to 6 months. The biological definition of T2DM was fasting blood glucose ≥ 1.26 g/L (7.0 mmol/L) after 8 hours with two measurements. T2DM was targeted to achieve a glycated hemoglobin (HbA1C) level < 6.5%. Sleep apnea was diagnosed by polysomnography and corresponded to a stop of air flow for 10 seconds or more. It was associated with oxygen desaturation ≥ 4%. Sleep apnea syndrome was defined as an apnea index > 5 (number of apneas during 1 hour of sleep) or an index of apnea-hypopnea > 10. Dyslipidemia corresponded to at least one of the three following biological assays: LDL-cholesterol ≥ 1.60 g/L (4.1 mmol/L) and/or HDL-cholesterol < 0.40 g/L (1 mmol/L) and/or triglycerides ≥ 1.50 g/L (1.7 mmol/L).

### 2.7. Statistical Analysis

We expressed quantitative variables such as weight parameters as mean and standard deviation (SD), and these parameters were compared using *t-*test. The correlation analysis of the % EBMIL at M3 and at M24 was constructed using the Pearson correlation test. The % EBMIL speeds were estimated by calculating the weight ratio per trimester, which is considered to be the best approach (e.g., % EBMIL operative time- (OP-) M3 = (weight at M3)/(weight at operative time)). % EBMIL speed comparisons were performed using Student's *t*-test. Finally a linear regression analysis was made in order to calculate a % EBMIL at M3 predictive of the success of this bariatric operation. The statistical software Prism version 6.0 (GraphPad Software, San Diego, California, US) was used for the analysis of the data. Statistical significance was set at *p* < 0.05.

## 3. Results

### 3.1. Patients' Characteristics

Between January 2007 and December 2010, two hundred forty-two consecutive patients had SG. One hundred fourteen patients were lost to follow-up or did not complete the entire follow-up with the four postoperative appointments (47.1%). Finally, one hundred twenty-eight patients were included, for whom all two-year follow-up data were available. Most of the patients were female (78.9% female : 21.1% male), with a mean age of 39.9 (11.0) years and a mean BMI of 49.3 (7.4) kg/m^2^ before SG.

The main comorbidity was sleep apnea (26.6%) and then hypertension (19.5%), T2DM (16.4%), and dyslipidemia (5.5%). Mean preoperative weight was 133.7 (21.0) kg, with a mean ideal weight based on the Lorentz formula of 59.6 (6.2) kg. All preoperative data are shown in Table 1.

### 3.2. Complications

Two cases of early gastric leaks and two cases of early gastric stenosis were reported. Thus, data concerning these patients were not taken into consideration, as the effect on weight loss can be significant.

### 3.3. Weight Loss (Figure 1)

**Figure 1:**
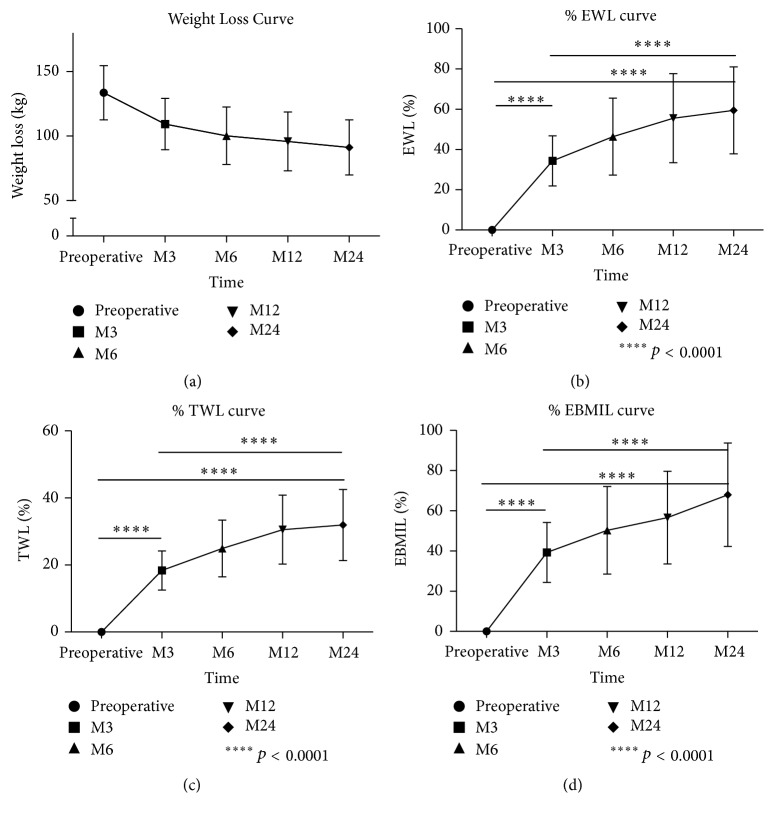
Evolution in weight parameters during 24 months of follow-up. M3: at 3 months; M6: at 6 months; M12: at 12 months; M24: at 24 months. (a) Weight Loss Curve before and after SG: preoperative and at M3, M6, M12, and M24. (b) % EWL curve before and after SG: preoperative and at M3, M6, M12, and M24. (c) % TWL curve before and after SG: preoperative and at M3, M6, M12, and M24. (d) % EBMIL curve before and after SG: preoperative and at M3, M6, M12, and M24.

For a large proportion of patients, weight loss was major during the first trimester.  Figure 1(a) shows that, after the first three months, weight loss continued until two years but with a slower aspect.  Figure 1(b) shows that % EWL was significant especially between surgery and M3, but also between surgery and M24, and M3 and M24. The same results were found concerning % TWL (Figure 1(c)) and % EBMIL (Figure 1(d)). Therefore, we focused on % EBMIL which is a good criterion of success for bariatric surgery.

### 3.4. Correlation of Excess Weight Loss at M3 and M24

**Figure 2:**
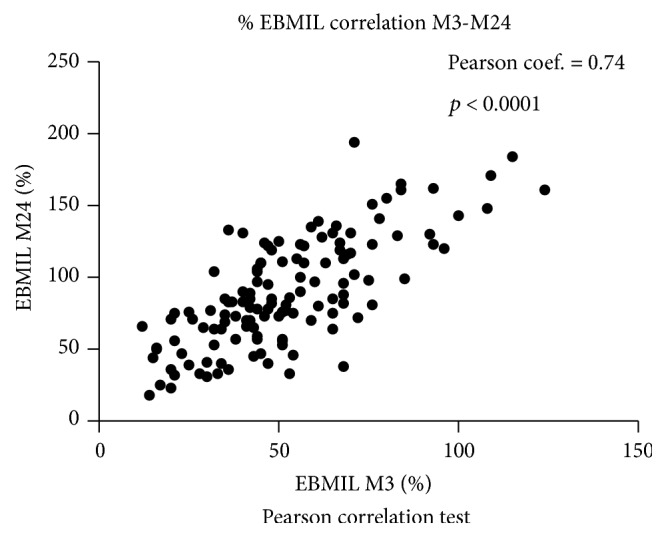
Excess Body Mass Index Loss (% EBMIL) correlation test between M3 and M24. EBMIL: Excess BMI Loss; M3: at 3 months; M24: at 24 months.

According to our clinical experience, we formulated the hypothesis that the % EBMIL at M24 was linked to the result at M3. Therefore, a Pearson correlation test was constructed and showed that the correlation between the % EBMIL at M24 and the early % EBMIL at M3 was statistically significant (*r* = 0.74; *p* < 0.0001; CI 95%) (Figure 2).

### 3.5. Comparison of Excess Body Mass Index Loss Speeds between Operative Time and M3 and Operative Time and M24 per Trimester

**Figure 3:**
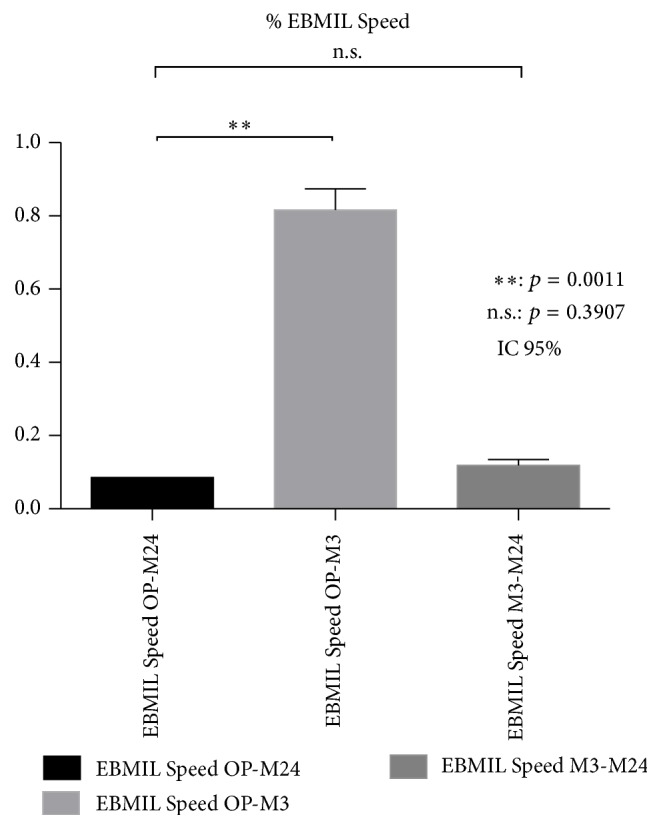
Comparison of Excess Body Mass Index Loss (% EBMIL) speeds between operative time-M3 and operative time-M24. EBMIL: Excess BMI Loss; OP-M3: time interval between operative time and 3rd month; OP-M24: time interval between operative time and 24th month; M3-M24: time interval between 3rd month and 24th month. EBMIL speeds are compared using the paired *t*-test.

As we previously explained, it was decided to express % EBMIL speeds using the ratio of weight at two time points, which is statistically a very acceptable expression of speed (e.g., (weight M3)/((weight at OP)). It appears that % EBMIL between operative time and M3 is statistically significant (*p* = 0.0011; CI 95%) whereas it is not significant between operative time and M24 per trimester (*p* = 0.3907; CI 95%) (Figure 3). This result suggests that patients lost most of their % EBMIL during the first three months after the operation until the final result estimated at M24.

### 3.6. Determination of Predictive Excess Body Mass Index Loss at Three Months

**Figure 4:**
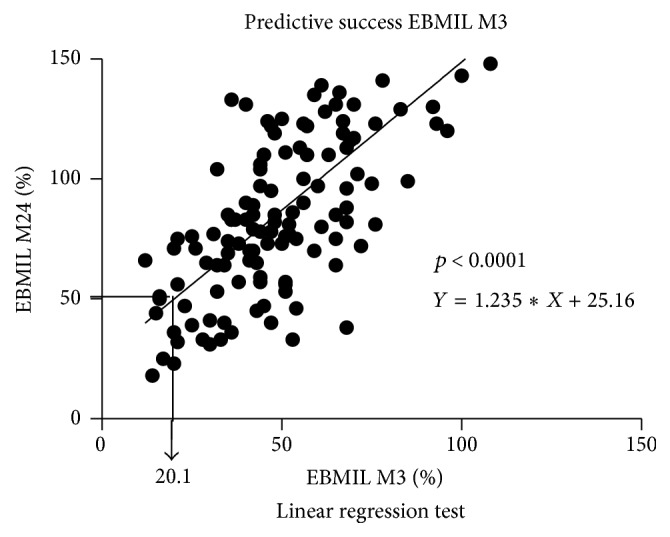
Determination of the success of predictive Excess Body Mass Index Loss (% EBMIL) at M3 (linear regression analysis). EBMIL: Excess BMI Loss; M3: at 3 months; M24: at 24 months.

Therefore, linear regression analysis allowed us to calculate the % EBMIL at M3 predictive of the success of SG. We found that a % EBMIL at M3 higher than 20.1% (*p* < 0.0001; CI 95%) was predictive of the success of the procedure (Figure 4).

### 3.7. Effect of Weight Loss on Resolution of Comorbidities

**Figure 5:**
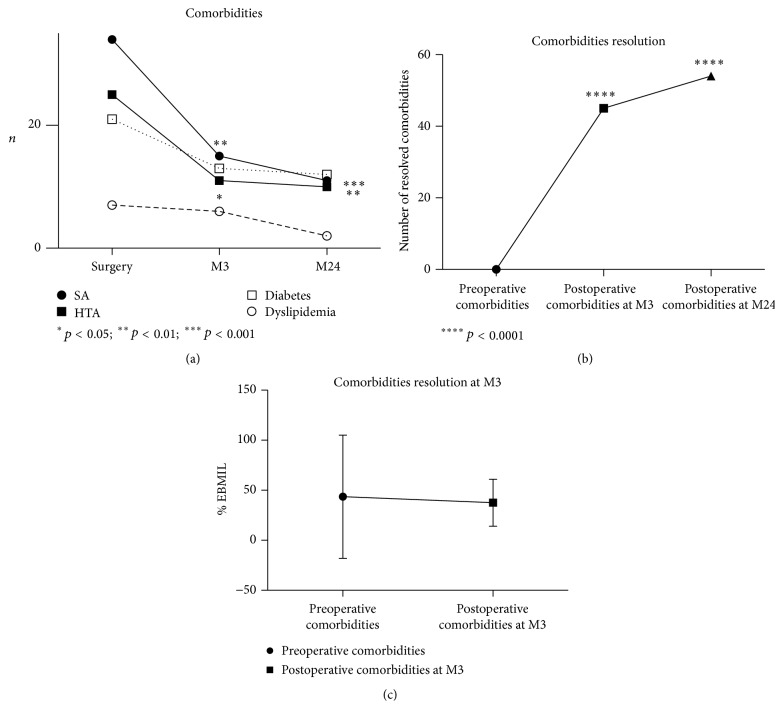
Evolution of comorbidities. (a) Evolution of comorbidities in preoperative time and three months and two years after surgery. SA: sleep apnea; HTA: hypertension; M3: at 3 months; M24: at 24 months. (b) Resolution of comorbidities at M3 and M24 for all comorbidities of 54 patients presenting with at least one preoperative comorbidity. M3: at 3 months; M24: at 24 months. (c) Relationship between Excess Body Mass Index Loss (% EBMIL) and resolution of comorbidities. M3: at 3 months.

Fifty-four patients presented with at least one comorbidity.  Figure 5(a) shows that, at three months and at two years after surgery, comorbidity rates had decreased and were particularly significant regarding sleep apnea and hypertension (*p* = 0.0039 and *p* < 0.001; *p* = 0.019 and *p* = 0.006, resp., at M3 and M24). For thirty-eight of the fifty-four patients all comorbidities were resolved at the end of follow-up. Considering the entire number of comorbidities among these patients, postoperative comorbidities decreased from the third postoperative month (*p* < 0.0001) until the two-year postoperative period (*p* < 0.0001) (Figure 5(b)). No significant difference in correlation to weight loss was observed (*p* = 0.11) (Figure 5(c)).

## 4. Discussion

The results of the present study show that % EBMIL at three months after SG is predictive of the success of the procedure if higher than 20.1%. We also show that % EBMIL speed in the first three months is strongly correlated with long-term results while this was not found in the following months. This analysis shows the determining role of the first trimester after surgery and the importance of multidisciplinary follow-up, which may lead to a positive long-term result. We believe that % EBMIL, according to the estimated ideal weight, should be calculated at all appointments in order to predict the long-term result. To our knowledge, this is the first study that determines that the early result of SG may be predictive of the final result based on % EBMIL.

Many studies have already demonstrated that other predictive factors can have an impact on % EBMIL in the long term. Size of bougie [11] and distance of resection from the pylorus [12] are the two main preoperative factors known to have an effect on weight loss result. Indeed, Abd Ellatif et al. recently demonstrated that small bougie size ≤ 36 F and leaving a short distance to pylorus ≤ 4 cm was associated with higher % EBMIL. Volume of the gastric reservoir has also been estimated in the postoperative period, but results remain discordant. Weiner et al. [16] reported that a lower gastric reservoir using a smaller bougie was associated with increased % EBMIL. However, Sánchez-Pernaute et al. [17] in 2007 did not find this relationship, but this study only concerned patients who had BPD/DS and not SG. Vidal et al. [13] recently described a new radiological method that showed a relationship between increased gastric reservoir and lower weight loss after SG.

Several predictive factors of success have already been established. Keren et al. [18] recently analysed postoperative lifestyle modifications and concluded that nutrition habits and physical fitness modifications were strongly associated with the success of the surgery. Other established predictive factors are preoperative weight loss, which is negatively associated with weight loss after surgery [19], the influence of the learning curve on % EBMIL efficacy (estimated at the 68th case) [20], the importance of postoperative follow-up on weight loss and improvement in comorbidities [21], age, race, and diabetes [22].

Nevertheless, our study has several limitations. First of all, as in many clinical studies, we excluded patients without full follow-up. Most of them did not reach postoperative year 2. However, only 10% of patients were definitively lost to follow-up; some experienced failure of the procedure while others with good results refused further follow-up. Hence, we did not exclude patients with untypical weight loss patterns and our results are representative of our whole population of patients. Secondly, we did not show results of % EBMIL at five years as recommended by the IFSO to estimate the success of a bariatric procedure. In fact, we were unable to retrieve data for all our patients as the majority of them had their SG procedure less than five years ago. The question of weight regain remains unresolved in this particular case. Moreover, complications were not taken into consideration, but this was not our main endpoint. However, there is a real possibility that complications could have an impact on patients' weight loss and % EBMIL as a consequence. We focused on early excess weight loss as a predictive factor of success, and many other studies have already estimated the impact of SG on comorbidities [23–26].

Our results could have a considerable impact on the care and management of obese patients. Indeed, early detection of failure of SG has led us to develop intensive follow-up protocols, with better patient management in terms of diet and sport activities. As suggested by Keren et al. [18], intensive follow-up protocols could help to lower the failure rate of SG.

Our results could also justify early performance of other procedures such as BPD/DS, RYBP, or re-SG. Cheung et al. [27] recently analysed 214 cases of revisional bariatric surgery following failed primary SG: their results showed a % EWL at 24 months of follow-up of 60 and 68% after conversion to RYBP and re-SG, respectively. These data confirm that a second procedure seems to have the same efficacy as a successful primary procedure, with a % EBMIL over 50% in the long term. For forty-four of our patients, SG was considered as a failure because of a % EBMIL of less than 50% after two years: a revisional procedure could thus be proposed to these patients.

## 5. Conclusion

Our study shows that a % EBMIL over 20.1% at three months is predictive of the success of this procedure, and it appeared that patients lost most weight during the first three months after surgery. However, further and larger studies are warranted to confirm these results with a longer follow-up of at least 5 years with the objective of improving patient management after sleeve gastrectomy.

## Figures and Tables

**Table 1:** Patients' characteristics at baseline.

Variable	*N* = 128
Age (years ± SD)	39.9 ± 11.0
Sex (female : male %)	78.9 : 21.1
BMI (kg/m^2^, mean ± SD)	49.3 ± 7.4
Sleep apnea (%)	26.6
Hypertension (%)	19.5
T2DM (%)	16.4
Dyslipidemia (%)	5.5
Preoperative weight (mean ± SD)	133.7 ± 21.0
Ideal weight (mean ± SD)	59.6 ± 6.2

BMI: Body Mass Index; T2DM: type 2 diabetes mellitus.
